# Reaching into the future

**DOI:** 10.7554/eLife.101739

**Published:** 2024-09-02

**Authors:** Raeed H Chowdhury

**Affiliations:** 1 Department of Bioengineering, University of Pittsburgh Pittsburgh United States

**Keywords:** sequential movement, eye movements, motor planning, sequential reaching, reaching, Human

## Abstract

When carrying out a sequence of movements, humans can plan several steps in advance to make the movement smooth.

**Related research article** Kashefi M, Reschechtko S, Ariani G, Shahbazi M, Tan A, Diedrichsen J, Pruszynski JA. 2024. Future movement plans interact in sequential arm movements. *eLife* **13**:RP94485. doi: 10.7554/eLife.94485.

Recently a friend introduced me to Beat Saber, a virtual reality game that involves using virtual swords to rhythmically slash through colored blocks that come streaming towards the player. After twenty minutes of flailing my arms around with minimal success, I gave up. My friend then passed the headset to his eight-month-pregnant wife who, after being helped off the couch, switched the difficulty to ‘expert’ and within seconds blew past my score with unbelievable grace and efficiency. Watching her, one thing became clear: while I had been focusing on how to slash the blocks nearest to me, she had already started to think about and prepare for the blocks in the distance as she slashed through those closest to her.

This ability to prepare future movements allows us to perform everyday tasks, like writing and washing dishes. However, many questions remain about how this continuous planning works in the brain. For instance, how many movements can the brain prepare in advance, and how malleable are these planned movements to change? Now, in eLife, J Andrew Pruszynski and colleagues – including Mehrdad Kashefi as first author – report new findings that help to answer these questions (Kashefi et al., 2024).

The team (who are based at Western University and San Diego State University) asked human participants to perform a task like Beat Saber (Figure 1A), where each individual had to reach towards a series of targets that appeared one after the other on a screen. However, unlike other sequential reaching tasks (Glaser et al., 2018; Zimnik and Churchland, 2021), this task also sometimes showed future targets beyond the upcoming one**,** allowing participants to plan multiple movements in advance, if they were able to.

**Figure 1.**
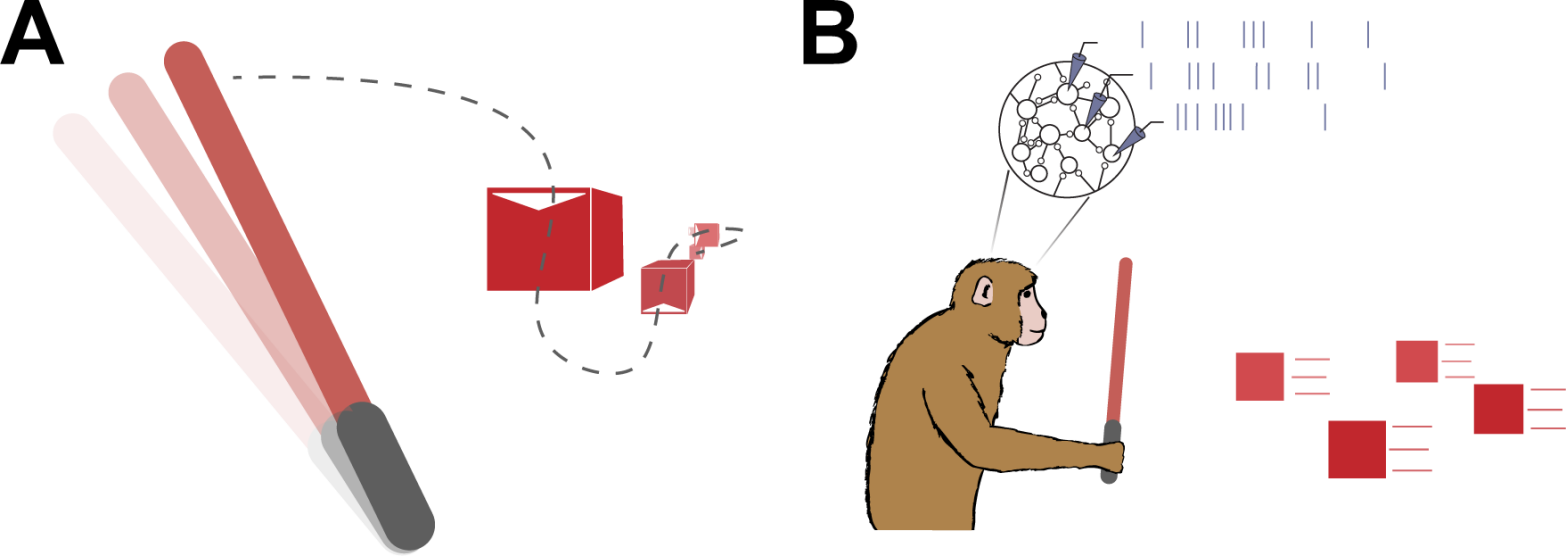
Planning a sequence of movements. (**A**) A schematic representation of the virtual reality game Beat Saber, during which the player must use a sword (left) to slash a stream of blocks (right) that are coming towards them – similar to the task Kashefi et al. used in their study. Based on results from Kashefi et al., knowing about future blocks influences planned movements (dashed line) and helps players to slash the boxes as they arrive. (**B**) A natural follow-up study would be to repeat the experiment in monkeys and use electrodes (triangles) to measure the spiking neural activity of their motor cortex (represented as a stream of vertical lines from each electrode) as they plan ahead.

Image credit: Raeed H Chowdhury; monkey illustration is by Carolina Massumoto.

Kashefi et al. found that participants could indeed plan at least two movements ahead. As they were given information on future targets, their movements became faster and more coordinated, with each movement smoothly transitioning into the next. It seemed that plans for movements in the distant future were not simply added to a queue, but instead modified movements in the near future to improve their efficiency.

Kashefi et al. confirmed this with a clever manipulation that involved making one of the targets jump, thus forcing the participants to re-plan their movements on the fly. When a target suddenly jumped to a new position on the screen, subjects were slower to respond to the jump if they already had information about the next future target. Overall, the results suggest that plans for future movements interact with and modify each other.

This interdependence between movement plans might suggest that short sequences of movements are planned together as a single trajectory, or ‘motor chunk’ (Ramkumar et al., 2016). In this scenario, once one chunk is completed, the subject moves onto the next, effectively planning one chunk – rather than one movement – at a time. However, Kashefi et al. show that this is not the case in their experiment. Participants transitioned smoothly and continuously between movements, without any discernible breaks that would indicate motor chunks. Furthermore, when a target jumped to a new position, participants were able to flexibly change individual movement plans to compensate, rather than having to re-plan a whole chunk.

In the brain, movement planning is accompanied by specific patterns of neural activity in the motor cortex, a brain region critical for skilled motions like playing a musical instrument (Strick et al., 2021). These patterns of neural activity help the brain prepare for an upcoming movement and are distinct from the activity patterns that control the actual movement (Ames et al., 2019; Churchland et al., 2010; Elsayed et al., 2016; Schimel et al., 2024).

One highly relevant study explored these preparatory neural patterns in monkeys reaching for two consecutive targets (Zimnik and Churchland, 2021). Like the study performed by Kashefi et al., the researchers found that monkeys did not prepare movements in chunks. Instead, the preparatory neural patterns that arose during the first movement prepared the monkeys to reach for the second target. However, while the study in monkeys suggested that sequential movements are prepared independently**,** Kashefi et al. went further by using trials with many movements and adding more targets to the planning horizon. In doing so, they showed that the preparation of future movements is in fact interrelated.

This difference between the two studies opens up a number of questions. For instance, what does neural activity look like during multi-movement planning tasks like the one conducted by Kashefi et al. (Figure 1B)? Also, what happens in the brain when plans for future targets interact, or when planned movements must change as targets unexpectedly jump to new positions?

While researchers have a reasonably good understanding of how individuals prepare single movements, much less is known about preparing movement sequences. The task and behavioral analysis undertaken by Kashefi et al. provides an excellent foundation upon which to build this knowledge. Until then, I will just have to continue practicing to get better at Beat Saber.
